# “Many people have no idea”: a qualitative analysis of healthcare barriers among Yazidi refugees in the Midwestern United States

**DOI:** 10.1186/s12939-022-01654-z

**Published:** 2022-04-11

**Authors:** Falah N. Rashoka, Megan S. Kelley, Jeong-Kyun Choi, Marc A. Garcia, Weiwen Chai, Hazim N. Rashawka

**Affiliations:** 1 Department of Nutrition and Health Sciences, University of Nebraska-Lincoln, 1700 N 35th Street, Leverton Hall, Lincoln, NE 68503 USA; 2 Department of Social Work, California Baptist University, Riverside, CA USA; 3 Department of Sociology, Syracuse University, Syracuse, NY USA; 4 Independent Expert Consultant, Lincoln, NE USA

**Keywords:** Healthcare disparities, Communication barriers, Refugees, Minority health

## Abstract

**Background:**

The COVID-19 pandemic has shed new light on inequities in healthcare access faced by immigrant and refugee communities. To address ongoing disparities, there is an urgent need for ecological approaches to better understand the barriers that hinder and resources that facilitate access to healthcare. This study investigates barriers to healthcare system access faced by Yazidi refugees in the Midwestern United States.

**Methods:**

Informed by the Interpretative Phenomenological Approach, three focus group meetings with a community advisory board were conducted between September 2019 and January 2020. The nine-member focus group included social workers, healthcare providers, and members of the Yazidi community. Meeting recordings were transcribed into English, coded for themes, and validated.

**Results:**

We describe themes related to specific barriers to healthcare access; analyze the influence of relational dynamics in the focus group; explore experiential themes related to healthcare access in the Yazidi community, and finally interpret our findings through a social-ecological lens.

**Conclusion:**

Community agencies, healthcare organizations, policymakers, and other stakeholders must work together to develop strategies to reduce systemic barriers to equitable care. Community representation in priority-setting and decision-making is essential to ensure relevance, acceptability, and utilization of developed strategies.

## Introduction

The Yazidi are a historically persecuted ethnic and religious minority group from northern Iraq [1, 2]. After a brutal attack on their homeland by the Islamic State of Iraq and Syria (ISIS) in August 2014, more than half a million Yazidi survivors fled to refugee camps. Many families relocated to the U.S., where Lincoln, Nebraska has become known as the ‘capital’ of Yazidis residing in the U.S. [3, 4]. Although there are some social benefits to residing in a sizeable refugee community, significant barriers to successful adaptation to U.S. society persist.

### Barriers to healthcare

Refugees often experience significant barriers that can impede access to healthy food, mental health services, and healthcare in their host country [5]. Individual and community experiences shape some barriers to care, while others are driven by organizational and system-level practices and policies.

#### Mental health and trauma

Traumatic experiences including war, genocide, terrorism, displacement, living in high conflict areas, and sexual abuse lead many refugees to experience adverse mental health effects, such as post-traumatic stress disorder (PTSD). After resettlement, the loss of social support, property, wealth, and occupation status can cause many refugees to suffer further traumatic effects such as depression, chronic stress, and severe isolation and loneliness [6–8]. These losses are often acutely experienced by elderly refugees, who must cope with severely reduced social networks and community ties [6, 9, 10].

#### Acculturation

For many refugees, adapting to the new healthcare environment in the U.S. is a challenge [11]. Differences between the healthcare systems in a refugee’s country of origin and their host country are often not intuitive [12]. In addition, cultural beliefs and perceptions can influence refugees’ desire to make use of certain healthcare resources, such as preventive services, mental health services, and treatment for conditions such as sexually transmitted infections and Alzheimer’s disease [13, 14]. Experiences of stigma and discrimination in the host country can tarnish perceptions of care providers and lead to a lack of trust that can damage the patient-provider relationship and cause refugees to avoid seeking health and mental healthcare [12, 15, 16].

#### Language

Refugees resettle with varying degrees of fluency in the language of their host country. Communication barriers between refugees and their healthcare providers pose one of the most limiting barriers to healthcare access [11]. While interpreters are frequently called upon to assist with healthcare delivery and may provide services in person or over the phone, many refugees dislike interpretation by phone [5]. Additionally, the presence of an interpreter does not guarantee that language and cultural barriers will be fully addressed. For example, interpreters may not speak precisely the same language or dialect as the patient [11]. Even when language barriers are overcome, refugee patients (notably women) may find it difficult to adequately express their physical symptoms in the presence of an interpreter [17]. Difficulties imposed by interpretation and language barriers are so consequential that it is not uncommon for refugees to utilize healthcare services only when they are extremely sick [5, 11, 18].

#### Access barriers

Medicaid eligibility requirements, costly healthcare, and lack of health insurance coverage lead many refugees to not seek healthcare services when it is necessary [11]. Moreover, when refugees can access health insurance through an employer, the policy often covers only the employee, leaving their family uninsured [11, 18]. Health coverage itself does not ensure access to care; many refugees do not have a reliable mode of transportation [5]. Public transportation systems operate on specific schedules and routes, which can be difficult for many refugees to follow, especially those with limited English proficiency [11, 19]. The high cost of transportation is another factor that keeps access to care out of reach for many [20]. Finally, those refugees who surmount the aforementioned barriers may encounter long waiting times for making an appointment and seeing medical specialists [21–23]. Refugees who are used to more timely care in their countries of origin commonly seek healthcare in emergency rooms as they can walk in and be seen without an appointment [5]. This approach results in higher medical bills and may interfere with the establishment of a medical home for regular and preventive care.

Given that approximately 20% of Americans are projected to be foreign-born by 2060 [24] and that global migration is projected to significantly increase in the coming decades [25], it is imperative that healthcare systems incorporate organizational strategies and care delivery practices to meet the needs of rapidly changing populations.

### Purpose

The purpose of this qualitative study is to gain insight into how Yazidi refugees experience facilitators and barriers to healthcare access. Specifically, we examine knowledge, beliefs, and behaviors about health and healthcare utilization; community-level relationships and resources, and environmental and contextual challenges and supports.

## Methods

### Qualitative approach and research paradigm

The social-ecological model conceptualizes the ways in which one’s environment influences health behavior [26, 27], and can provide insight into how individual, interpersonal, and sociocultural factors combine to produce health disparities for marginalized refugees [28]. In addition, the use of qualitative methodologies can help researchers gain insight into real-world implications of complex health-related phenomena in vulnerable populations [29–31]. Situated in the interpretivist research paradigm, this study was informed by the Interpretative Phenomenological Analysis approach (IPA), the aim of which is to “explore in detail individual personal and lived experience and to examine how participants are making sense of their personal and social world” [32]. This approach was selected due to its view of reality as both individually and socially constructed, and because it takes into account the interaction between researcher and participant in creating knowledge. Focus groups can be especially useful in the study of health disparities as the interpersonal dynamics may lead participants to share more personal insights [33, 34]. Some researchers express caution regarding the use of focus groups in IPA due to the potential that a) participants may hesitate to express themselves freely [35] and b) it may be difficult to give appropriate consideration to both the experience of the individual and the experience of the group [35]. However, others have noted that when using focus groups in IPA research, “certain insights appeared to arise not in spite of, but because of, the shared experiences and understandings of the group” [36] and that “it might well be that the best use of a focus group when being used with an IPA *approach* is to use the group dynamic as a tool to enhance personal accounts” [37]. This paper follows the framework provided by Phillips, Montague, and Archer [38], which incorporates into the iterative data review process an analytic strategy to investigate group effects on the focus group data, followed by a re-evaluation of the data. To that end, this paper begins with an initial analysis of themes related to specific barriers to healthcare access; proceeds with an analysis of relational dynamics in the focus group; explores experiential themes related to healthcare access in the Yazidi community, and finally interprets our findings through a social-ecological lens.

### Researcher characteristics and reflexivity

The IPA approach recognizes that the researcher cannot be completely objective, but rather that their experience and perspective play an active role in the interpretative process [32, 39, 40]. MK is a white U.S. native with a doctoral degree in health promotion research and limited prior knowledge about the Yazidi. FR is a Yazidi community member with an educational background in nursing (BSN) who was a graduate student at the time of the study. FR had previous experience interacting with the Yezidi community in the context of his work in the community service clinic at the local public health department. His goal in his later position as a graduate researcher was to build on what he observed from that experience to better understand barriers that affected healthcare access disparities for the Yezidi community. FR’s position as a trusted and respected member of the community facilitated participant recruitment and enabled rich dialogue during the focus groups. During the focus groups, he protected his role as a researcher by following the discussion guide and refraining from inserting his own experiences and perspectives into the discussion. HR is also a member of the Yazidi community with a medical degree. The researchers’ combined backgrounds allowed for an overall process that was attuned to the demands of both cultural appropriateness and research integrity.

### Context

This study takes place in the context of the development of a community health education project. In the Interpretative Phenomenological Approach, a smaller sample size allows for a deeper and richer understanding of experience [41]. Three meetings with a focus group of nine members were conducted to facilitate discussion around perceptions and experiences of Yazidi refugees regarding access to healthcare services and resources. Recruitment was conducted by a researcher who is a member of the Yazidi community (FR). We employed purposive sampling to identify and recruit participants with extensive knowledge about Yazidis’ experiences in obtaining healthcare. Leask, Hawe, and Chapman [42] suggest that natural groups - in which members may have met before - may be preferable for focus groups exploring sensitive topics. Focus group participants in this study were members of a community advisory board established for the community health education project. We began by contacting community-based organizations and health clinics who serve large refugee populations. They identified representatives familiar with Yezidi community needs to participate in the focus groups. We also identified and recruited unaffiliated Yazidi community members who were well-connected within the community and familiar with common experiences and needs. Participants were approached by text message and email. Of the organizations invited, two were unable to participate. Of the individuals invited, two were unable to participate due to time conflicts.

### Ethical issues pertaining to human subjects

This study was reviewed and certified exempt by the IRB of the University of Nebraska-Lincoln.

### Data collection methods

Focus group meetings took place in-person. The first focus group discussion in September 2019 covered barriers and facilitators to healthcare access. In the second and third meetings in October 2019 and January 2020, the identified themes were reviewed and further refined. Discussion guides were sent to participants by email several days before each meeting. They included eight questions regarding barriers to healthcare, experiences with the healthcare system, and recommendations for education. During the first meeting, participants were fully apprised of the study’s aims and protocol prior to providing written informed consent. Each meeting was audio-recorded and lasted approximately 2 h. FR and MK conducted the meetings. FR led the meetings, while MK took notes and asked follow-up questions. FR’s fluency in Kurdish Kurmanji, Arabic, and English languages enabled participation in participants’ language of preference. Participants were offered $25 as compensation for their time.

### Participant characteristics

Nine focus group members agreed to participate in this study. Average participant age was 39.44 (± 3.91) years, and 56 % of participants were male. Education level varied: 55% were college graduates, 22% were high school graduates, and 22% had less than a high school education. Concerning language proficiency, 55% were native Kurdish Kurmanji speakers who could speak another language. Additional participant characteristics are outlined in Table 1.

**Table 1:** Characteristics of Focus Group Participants and Research Question

Participant Characteristics	
N	9
**Age (years)**	39.44 ± 3.91
**Gender**	
Male	5 (56%)
Female	4 (44%)
**Education**	
Less than high school	2 (22%)
High school graduate	2 (22%)
College graduate	5 (55%)
**Language**	
English	7 (78%)
Kurdish Kurmanji	5 (55%)
Arabic	5 (55%)
**Number of children**	
None	4 (44%)
1–2	1 (11%)
3–4	2 (22%)
≥ 5	2 (22%)
**Occupation**	
Refuge agency/culture center staff	2 (22%)
Healthcare provider	2 (22%)
Community member with LEP	2 (22%)
Interpreter	1 (11%)
Social worker	1 (11%)
Social worker/Interpreter	1 (11%)
**Agency of participants**	
Refugee Resettlement Agency	2 (22%)
Federally Qualified Health Center	1 (11%)
Public Health Department	1 (11%)
Refugee Community Cultural Center (RCCC)	2 (22%)
RCCC with program for women	2 (22%)
Refugee community members (unaffiliated)	2 (22%)

### Data processing

First, the audio recordings of the focus group meetings were translated into English and transcribed within 1 week after each session, and initial themes were coded by FR and HR and reviewed by FR, HR, and MK. Second, QSR NVIVO version 12 (QSR International Pty Ltd., Melbourne, Australia) was used to separately identify emerging themes. The results of the manual and computed initial thematic analyses were compared. Connected themes were sorted into clusters [43]. Next, interactive dynamics within the focus group were examined, clustered, and modeled by FR and MK to inform further analysis of the data [38], leading to the development of experiential themes. Audio recordings were stored in a password-protected computer and participants were given number IDs in the transcript and manuscript to protect participant identity.

The research team applied multiple approaches to enhance the validity of the study. First, data source triangulation was applied for data collection, which included perspectives from social workers, healthcare providers, interpreters, cultural center members, and Yazidi refugee community members [44]. Second, two investigators (FR and MK) attended all focus group meetings to triangulate multiple observations and conclusions regarding focus group outcomes [45]. Third, the English translation of participants’ contributions in a language other than English was validated by a second person (HR) fluent in their native language and compared to findings from the broader literature [46]. Two researchers coded the transcripts separately, and three conferred on their findings and resolved differences through discussion and consensus. Finally, two rounds of synthesized member-checking focus groups [47, 48] were conducted to validate themes related to healthcare access barriers. Participant recommendations were incorporated into the final themes.

## Results

### Barriers to healthcare access

**Fig. 1:**
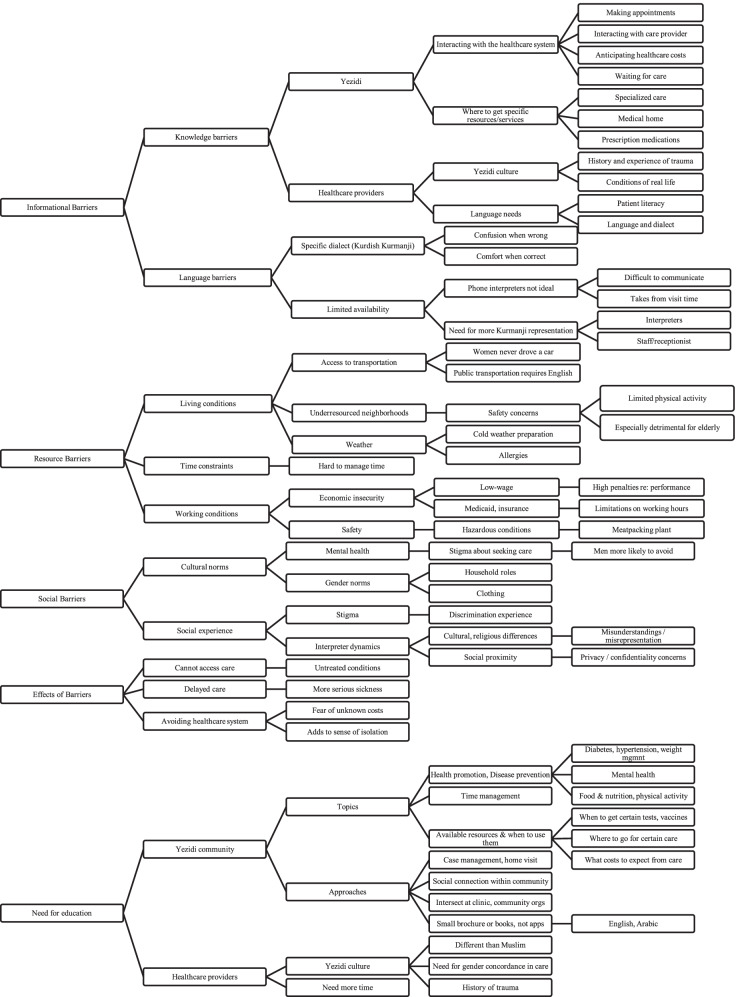
Barriers to healthcare experienced by the Yazidi community

Five superordinate themes on barriers to healthcare access were initially identified: informational barriers, resource barriers, social barriers, effects of barriers, and a need for education (see Fig. 1).

#### Informational barriers

The superordinate theme, *informational barriers*, describes barriers to healthcare access related to the accuracy and availability of information relevant to healthcare access. Participants noted knowledge gaps for community members, including how to navigate the healthcare system and where to go for care. One participant noted, “Yazidi community don’t know where to go, I did that several times, I am an example” [Participant 1]. He recounted that when he took his wife to a hospital to deliver a baby, he was told he was at the wrong hospital; it was a facility in the same hospital system but a different location. Participants described a common experience of confusion due to receiving unexpected medical bills from places other than the healthcare facilities they visited. Lack of clarity around medical billing leads to significant concerns about how unexpected costs could affect families; the same participant shared, “I got a bill from Kansas state, another bill from [hospital], and another one from emergency; I got a hundred bills I don’t know how to figure out, how to pay it.”

For healthcare providers, participants noted significant knowledge gaps about Yazidis’ cultural background and language needs. In addition to gaps in knowledge, the presence of language barriers was a significant theme: “language barriers prevent the refugees’ community from accessing healthcare, starting from making an appointment until medication prescription” [Participant 9]. Participants who worked as medical interpreters noted that often, the interpreters called to assist with Yazidi patients do not speak the correct Kurdish dialect – Kurmanji – which leads to errors in translation. Participants agreed that it was important for interpreters to be from the Yazidi community since they would be familiar with the patient’s language and culture, which would facilitate communication and make patients more comfortable. However, others noted drawbacks to having an interpreter from the same community. A patient’s social proximity to a Kurmanji-speaking interpreter could inhibit communication and introduce concerns about patient privacy and confidentiality. They noted this concern might be especially relevant for medical visits of a sensitive nature, such as reproductive health, mental health, or a terminal illness.

#### Resource barriers

*Resource barriers* describe how limitations in both tangible and intangible resources impede access to and opportunities for healthcare. Resource barriers were clustered into themes related to living conditions, time constraints, and working conditions. Participants pointed out that Yazidi refugees are relatively new to the United States and that most women did not drive a car in their country of origin. In Yazidi families, men usually work during daytime hours while women stay home. This arrangement can be especially difficult for families who live in areas with limited access to public transportation. One participant noted, “Majority of refugees and immigrants live in an isolated neighborhood where public transportation is not reaching, living in poor properties with no essential services and lacking green [recreational] area” [Participant 5]. Participants further noted that residing in under-resourced neighborhoods can be particularly detrimental for those with chronic diseases for whom regular exercise and outside activities are very beneficial. They described how not having an area to walk or do exercise has contributed to higher rates of overweight and obesity in the Yazidi community, increasing the likelihood that chronic diseases will become even more prevalent.

Regarding time constraints, one participant noted, “right now, I don’t have enough time to do all tasks that I need to do to my family and myself” [Participant 3]. Another added, “our people are out of schedule in their life. They don’t know how to organize their time... they miss many appointments because of lacking to scheduling their time.” [Participant 1]. Participants also described how economic and safety concerns in the workplace impeded their access to healthcare. Many refugees work in low-wage jobs that do not offer health insurance and are not able to afford or obtain private health insurance coverage. As an example, one participant elaborated on the impact of insurance availability: “many of Yazidi community don’t have insurance. They are not qualified for government insurance and at the same time working at low wages jobs which not offering insurance” [Participant 5]. Without Medicaid coverage, participants shared they would be unable to afford healthcare services. However, government-funded health insurance programs require refugees to have guaranteed income below the federal poverty level to be eligible, so those who qualify for Medicaid risk losing coverage upon earning more income. Yet, when refugees lose Medicaid, they are unable to afford other health insurance due to working in low-wage jobs. Furthermore, insurance policies require insured refugees with chronic disease to visit their providers every 3-6 months to continue eligibility. Participants noted that these compounding barriers lead many to avoid seeking needed healthcare.

#### Social barriers

*Social barriers* include cultural norms and social experiences that can reduce healthcare access. Participants noted that gender-related cultural norms influenced access to and use of healthcare services to a great degree. They also noted that women commonly prefer to have gender-concordant interpreters, especially for sexual and reproductive health concerns. One participant described the reaction of his wife when a female doctor and interpreter were not provided: “My wife refused to be seen and we get back to home without getting treatment” [Participant 9].

Participants shared that Yazidi refugees, particularly men, rarely pursue treatment for mental or behavioral health issues, including stress, depression, and Alzheimer’s disease. There is a high prevalence of negative mental health outcomes due to exposure to war and genocide. The 2014 ISIS attack was such a culturally devastating event that the date is commemorated each year as a day of mourning for the Yazidi community. One of the participants said that they expected to constantly suffer from depression, anxiety, and stress due to the ISIS attack. However, participants stated that shame and stigma prevent Yazidi refugees from sharing mental health concerns: “Many people don’t want share or see other people watch them when they go to the counselor or providers for the mental health clinic” [Participant 4]. For those who do seek out mental healthcare, it can be difficult to find culturally appropriate services. Some participants noted that the presence of interpreters can pose barriers to seeking mental healthcare services.

Several participants stated that social isolation is an additional barrier, especially for the elderly during the winter. Many Yazidi grew up in a social environment; after relocating to the U.S., they may spend long periods isolated at home after losing their social ties. Many refugees experience social discrimination based on cultural expressions such as wearing traditional clothing or speaking their native language: “Yazidi older adult wear Yazidi traditional clothes which cause to them some kind of discrimination to access the care they need” [Participant 9]. Such experiences lead many to prefer to stay home to avoid discriminatory encounters.

Negative experiences with medical interpreters are an additional social barrier that can detract from the healthcare experience. One participant described an acrimonious encounter with an interpreter who was speaking the Sorani dialect, not the Kurmanji dialect spoken by Yazidi:

“I told the interpreter, ‘don’t be rude, I don’t understand what you say.’ He said while got mad at me, ‘it’s like you don’t want to talk with me, and you don’t want me to be your interpreter.’ I respond, ‘first, you speak different accent than what I do speak, and second, it’s hard to understand at hospital when there are on phone interpreter instead of face to face.’ I asked the hospital staff to replace him and bring one who speak Kurdish Kurmanji and understand our culture. Also, if they could bring face to face interpreter that would be great because it’s very difficult to explain your exact pain through the phone” [Participant 5].

#### Effects of barriers

The superordinate theme, *effects of barriers,* summarizes the ways in which informational, resource, and social barriers impact access to healthcare. In cases in which the barriers cannot be overcome participants are not able to access care, which can lead to conditions remaining untreated and becoming exacerbated. In other instances, the barriers lead to delayed care, which can result in more serious sickness or worsening of conditions. Some barriers lead people to avoid seeking care as much as possible, which can add to feelings of disconnectedness and isolation.

#### A need for education

The superordinate theme, *a need for education,* describes how participants looked to the education of community members and healthcare providers as a solution to many of the challenges imposed by the previously mentioned barriers to care. Participants provided recommendations on topics that would be beneficial for the Yazidi community, and suggested possible approaches for providing such education, including that, “it will be beneficial if there is some kind of guideline to make sure [refugees] know which kind of services they would get from a particular place they went to go” [Participant 9].

All participants agreed about the need for healthcare providers to be knowledgeable about the culture-specific challenges faced by the Yazidi community. In particular, they felt that education and training around Yazidi religious beliefs, cultural norms, and language needs – and how they differ from those of Muslim patients - would help providers be better prepared to meet the unique needs of Yazidi refugees in their care.

### Group dynamics

**Fig. 2:**
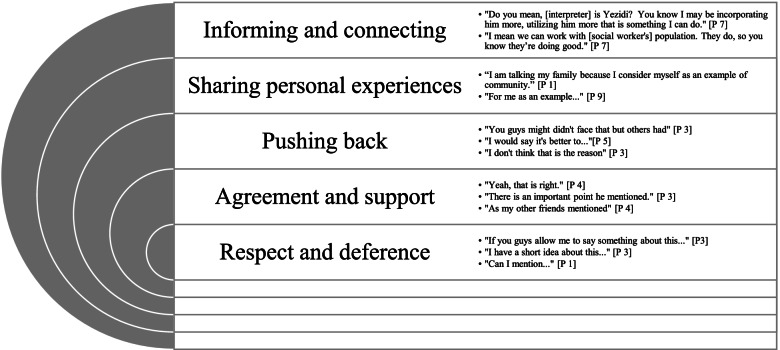
Focus group interaction analysis

Our analysis of relational dynamics between the focus group members led to the identification of five consistent forms of interaction that influenced the content and direction of the discussion. These themes and exemplary quotes are shown in Fig. 2.

#### Respect and deference

Interactions between focus group members were characterized by respect and deference. When sharing their own ideas or adding to a topic, participants politely interjected, sometimes even to the point of self-effacement. Overall, the group dynamic and interactional relationships in the initial and validation focus group meetings reflected that participants held each other in high regard.

#### Agreement and support

In many instances, participants expressed agreement with each other and support for each other’s ideas and experiences. These interactions seemed to foster an environment that led participants to feel comfortable contributing their perspectives.

#### Pushing back

Disagreement is common in any group of people who have varying experiences. Perhaps due to the presence of a supportive environment, participants in this study seemed to speak up when such disagreements came up. The expression of dissenting opinions allowed for a more nuanced analysis of the discussion; the presence of multiple points of view more fully informed our interpretation of the data.

#### Sharing personal experiences

Participants showed trust in each other by sharing personal experiences, including emotional issues and personal challenges when navigating the healthcare system. The sharing of personal stories throughout the discussion indicated that participants saw themselves as exemplars of their community’s experiences related to healthcare access.

#### Informing and connecting

Participants were included in this focus group due to their familiarity with the Yazidi community and its healthcare-related needs. The different professional and experiential backgrounds of the participants produced a robust discussion in which participants uncovered new and helpful resources and ideas from each other.

### Experiential themes

After re-evaluating the focus group transcript in light of the analysis of group dynamics, more experiential themes emerged from the data. These are outlined in Table 2 along with exemplary quotes.

**Table 2:** Experiential themes and exemplary quotes

**Primacy of language**	
	“The first [barrier] might be language.” [P1] “Yes, language, language, and language – the big problem that we all face.” [P1] “One of the biggest issues is the language barriers between providers and clients.” [P4]
**Contrast and tension**	
Before and after	“Everything is different.” [P3] “Might be the reason because we come from different country, different culture so we don’t know a lot about the process here in the U.S.” [P1] “I think they need to know about the Yezidi culture and history because our medical history is reflecting our history in Iraq.” [P1]
Caught in-between	“We are here and we still asking, and we don’t know, so what about people?” [P1] “I spend 3 years with military, I know some cultural stuff with American people but we still surprising me all this detail here. So, imagine people never seen anything like that.” [P3]
Negative perceptions / experiences impede care	“The process took between 12 and 13 h until I got treatment at emergency room. So, this point makes people hate getting insurance or even getting to doctors and seeking health care.” [P1]
**Precariousness**	
Uncertainty / Not knowing	“They don’t know a lot of information about health insurance. They don’t know how to apply. And even if they know how to apply, they don’t know how much they will pay, how much it will cost them. This is something our community afraid from it. They are confused about it.” [P1] “For example, I am working at [major retailer], and if I got late one minute, they will put one point on my record. My wife doesn’t have driver license and I have three kids. How we will go to appointment if we have one?” [P5]
Overwhelm	“Right now, I don’t have enough time to do all tasks that I need to do to my family and myself.” [P3] “Maybe you can’t see stress on my face, but probably I have stress inside due to the war which affected all my life. That happens to many Yazidi when they survived from genocide on the 2014.” [P1]
Financial instability	“We are new in this country and many of us have kids and some have chronic disease like me. So, when I got work just 8 h more than 20. Next day, I see they send me email saying your Medicaid stopped. You can’t work and try to depend on yourself.” [P5] “Many people don’t work more than limited time because they are afraid of losing Medicaid benefits for parents.” [P1]
**Adaptation and transition**	
Importance of education	“The system over all is good but we need transition. People need to know how to use that and they need education.” [P3] “Client should be educated in the same times, providers should know a little bit about the client culture, services he need.” [P1]
Importance of social connection	“Yezidi community is like a net, is all connected together.” [P3] “I know two elder people got back to Iraq because of social isolation; the community in Lincoln is so closed and we grow up in a social environment.” [P9]
Importance of trust	“I don’t trust private insurance.” [P9] “People don’t trust nurse due to the bad experiences with nurse work at our previous country.” [P9] “Health care provider need education about those barriers and how they can build a trust relationship with the client to get the health services they need.” [P4]
Doing the best you can	“We need to adapt with the situation. We need to arrange our time; if you don’t go to gym you can run; if you don’t want to run, you can do some exercise at your home. But you need to arrange your time, and because of transition and because of a lot of new things in our life.” [P3]

#### Language

When asked directly about barriers to healthcare, language was consistently the foremost response. As one participant put it, “language, language, and language – the big problem that we all face!” [Participant 1]. The theme, *primacy of language,* stands on its own, conveying how the availability of culturally and linguistically appropriate services (or lack thereof) has a significant impact on the lives and livelihoods of Yazidi refugees, and very notably so in the context of obtaining healthcare.

#### Contrast and tension

The superordinate theme, *contrast and tension,* focuses on the feeling of being between two worlds; in one sense, participants described feeling caught somewhere between a ‘before’ life in Iraq and an ‘after’ life in the U.S. They described how people often applied knowledge and assumptions from their ‘before’ life to their ‘after’ life, and how the disconnectedness of core elements of their identities from their new lived realities could lead to stress in daily life and miscommunications in healthcare. For the participants in our focus groups - especially those who worked in human and social service organizations and were themselves Yazidi – the focus group discussion itself seemed to reveal a sense of disillusionment at the realization of a tension between a self-perception of being a topical expert and cultural liaison, and a reality of still feeling constrained by many of the forces that hold less-acculturated community members back. Finally, participants described a clear tension between what people expected from the healthcare system and what they experienced – and that this tension led to negative perceptions and emotions around seeking care. This disconnect between expectations and reality was often rooted in assumptions grounded in the ‘before and after’ theme.

#### Precariousness

The superordinate theme, *precariousness,* centers on the sense of existing at a tipping point, and the feeling that not being able to maintain a delicate balance may lead to disaster. Participants’ descriptions of decisions and situations encountered in daily life revealed lives lived at the mercy of policies and systems of which community members typically have little knowledge, and over which they have no control. Underlying these descriptions seemed to be a persistent uncertainty driven by both a lack of the knowledge needed for success and a lack of any control over outcomes. Participants’ descriptions of their own and others’ experiences conveyed how the cumulative and persistent stresses of daily life contributed to an ongoing overwhelm. Many Yazidi refugee families rely on Medicaid for health benefits, but to remain eligible must limit the number of hours they work each week. The potential loss of health coverage for incremental increases in income led to a sense of hopelessness that they were unable to care and provide for their families as they wanted. One participant recounted that he had requested his wife not take him to the hospital if he becomes sick because he preferred to die at home so that his family did not receive a large medical bill: “it’s better dying at home than getting sixteen bills from different places” [Participant 1].

#### Adaptation and transition

The final superordinate theme of *adaptation and transition* shows the value participants placed in overcoming obstacles to thrive in their new environment. As in our initial analysis, education was an important theme related to participants’ experiences within the healthcare system and the wider world. A combination of education for community members and healthcare professionals would reduce stress, increase access, and lead to improved health outcomes. Also important for successful adaptation is a sense of social connection. Several participants described a sense of social connection and togetherness within the Yazidi community. One participant described how two elderly people returned to Iraq due to social isolation, suggesting that the sense of connectedness was influenced by ties to the broader community as well. A theme related to the importance of social connection is the importance of trust, especially as it applies to level of trust in healthcare systems and providers. Negative experiences that build distrust – both past and present - influence the likelihood that people will seek out the services they need. Participants noted that actively building trust is one step providers can take to support Yazidi refugees still transitioning to their new environment. In light of the number and the weight of the obstacles shared and discussed in this group, ‘doing the best you can’ emerged as a mindset that seems to serve the community as they transition to a new environment and a new life. As one participant noted, “if you don’t go to gym you can run; if you don’t want to run, you can do some exercise at your home.” This idea of making progress with available resources may support the process of cultural transition as it emphasizes the importance of personal agency even (and perhaps especially) in the context of precarity.

## Discussion

A recurring theme throughout the focus groups was informational barriers. Participants desired knowledge about many topics, including cultural norms in the United States, available community resources, how to access and navigate healthcare, what to expect when accessing care, and how and when to obtain over-the-counter medications. These findings are consistent with those of previous studies [5, 12, 21, 23, 49, 50]. Knowledge gaps in these areas had detrimental results: reliance on emergency rooms rather than the establishment of medical homes; mistrust of the healthcare system; and delaying healthcare or avoiding it altogether.

This study finds language and dialect barriers to be another central theme. This finding is consistent with previous studies which have found language to be a crucial barrier before, during, and after engagement with the healthcare system [10, 11]. Professional medical interpreters play an essential role in health communication and can improve patient experience and recovery [51–55]. Focus group members described significant benefits to having a medical interpreter from the same community but noted that privacy and cultural sensitivity concerns can diminish those benefits. This study supports findings that show gender-concordant interpreters may better facilitate communication for refugee women with limited English proficiency [6, 17].

The need for dialect concordance was a unique finding of this study. Extant research on the influence of regional dialect differences within language groups for access to and use of healthcare services is lacking [51–53].

Focus group members noted that most Yazidi refugees grew up in a very social environment and that feeling culturally isolated from the broader community can be very detrimental, especially for older adults. Consistent with other studies, participants reported that members of the Yazidi community often face a choice between low-wage employment or health insurance for their family through Medicaid [9, 56]. This arrangement effectively fences people into poverty due to fear of losing health insurance or compels them to pay for insurance plans that negate the financial gains of full employment at low wages. One neighborhood in which many Yazidi refugees reside is not well connected to public transportation (consistent with [5, 11]), and many Yazidi adults do not drive cars.

### Social-ecological interpretation and implications

**Fig. 3:**
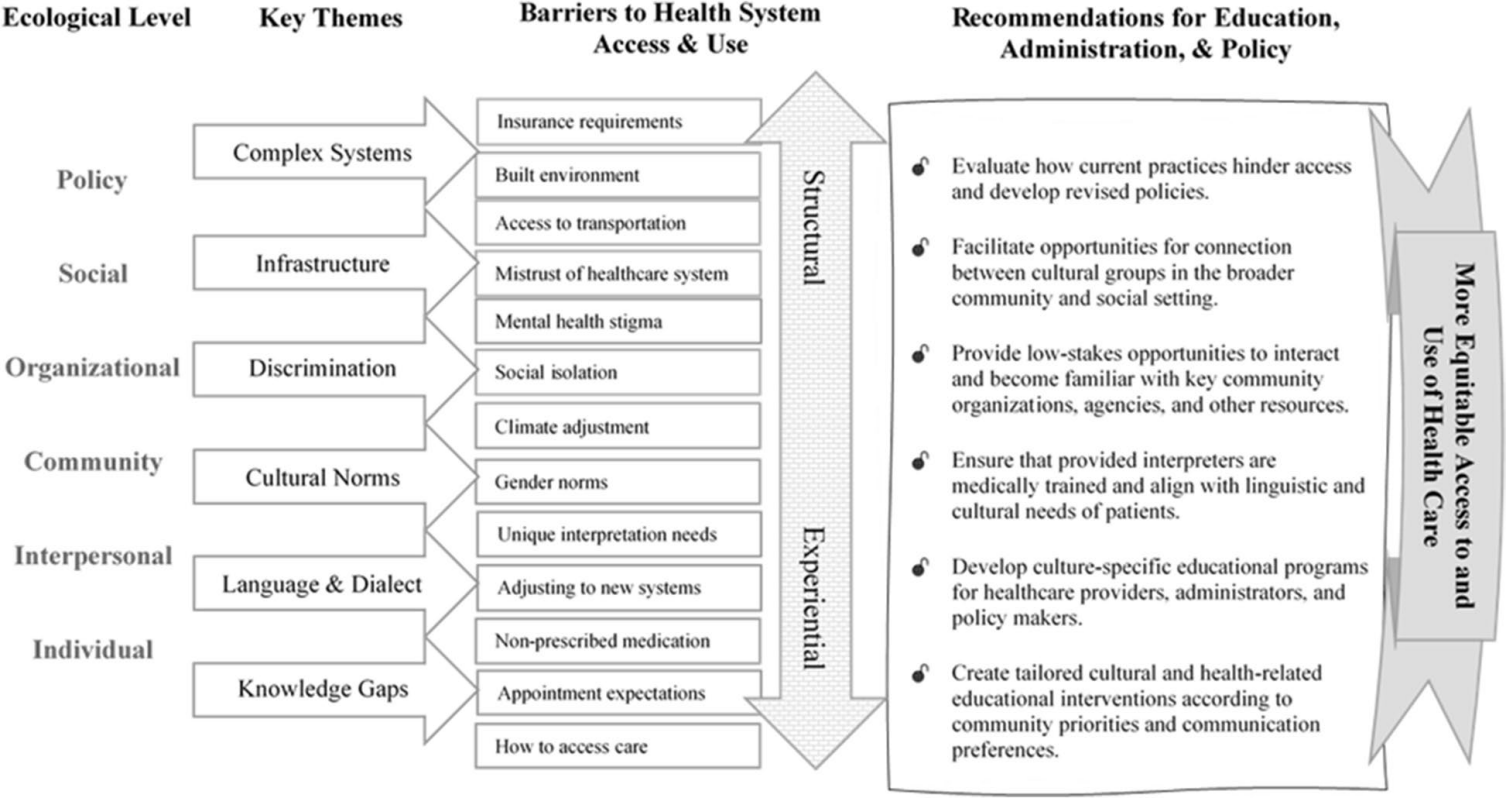
Yazidi focus group key findings and recommendations

Multilevel, interprofessional, culturally situated, and community-engaged approaches will be required to enable equity in healthcare access, opportunity, and experience for Yazidi refugees [57]. Based on the findings of this study, we outline a few potential strategies for educators, administrators, and policymakers (see Fig. 3).

#### Create tailored cultural and health-related educational interventions

A recurring theme throughout the focus groups was a need for more accessible, understandable, and actionable information about many topics including cultural norms in the United States, available community resources, how to access and navigate healthcare and insurance systems, what to expect when accessing care, and how and when to obtain over-the-counter medications. One approach to developing tailored educational interventions is to establish a community cultural advisory board to inform and review materials for cultural and linguistic appropriateness [58, 59]. Advisory boards play a critical role in the identification of community challenges and educational priorities.

#### Develop culture-specific educational programs

This study suggests a need for *culture-specific* training for care providers. Participants noted that Yazidi language, cultural customs, dietary habits, religious beliefs, and other areas that influence health behavior and healthcare needs are often overlooked. In the case of the Yazidi community, many suffer from post-traumatic stress disorder (PTSD), depression, and anxiety as a result of the 2014 ISIS attack in Sinjar, Iraq [60]. This study supports recommendations for cultural sensitivity training for healthcare providers who work with refugees [61], as providers who are not culturally knowledgeable may be unable to provide proper care [62].

#### Ensure that interpreters align with the linguistic and cultural needs of patients

The need for dialect concordance was a unique finding of this study. Participants noted that most medical interpreters called upon to assist Yazidi patients spoke the Sorani dialect, whereas the majority of Yazidi speak the Kurmanji dialect. Differences in pronunciation and terminology lead to frustration, anxiety, miscommunication, and inaccurate diagnoses; such complications have become especially consequential during the COVID-19 pandemic [63]. This finding highlights a need for healthcare providers and interpreters to be cognizant of and able to address the language and dialect needs of their patients.

#### Provide low-stakes opportunities to become familiar with key community resources

In addition to a need for information, there is also a need to build connections and trust. One approach to address this need is a cross-sector collaboration by community agencies and other partners to develop informal opportunities for community members to connect and become familiar with the key people, programs, and resources available in the community so that disadvantaged and marginalized community members know how to access the right resources at the right time.

#### Facilitate opportunities for connection between cultural groups in the community

Participants noted that most Yazidi refugees grew up in a very social environment and that feeling culturally isolated from the broader community can be very detrimental, especially for older adults. Weather is a contributing factor to the sense of isolation, as many Yazidis are not used to cold weather and prefer to stay at home during the winter. Participants also reported that experiences of discrimination in the healthcare setting and the broader community are not uncommon. Facilitated opportunities for connection between the Yazidi community and other groups may boost feelings of mutual identification and belonging. Increased access to practical knowledge and cultural insights may improve community members’ confidence to interact with the healthcare system and improve healthcare experiences.

#### Evaluate how current practices hinder access and develop revised policies

In addition to developing educational approaches and creating opportunities for connection, administrators and policymakers must prioritize policy improvement to address the hurdles over which community members have no control. Systematic reviews have found community health worker interventions to be effective in improving health behavior and outcomes and cost-effective to integrate into healthcare delivery for disadvantaged and marginalized groups [64–67]. The institutionalization of policies to train and support bilingual, gender-concordant community health workers may help address gaps in interpreter availability and the need for culturally appropriate care. However, trained community health workers may encounter challenges related to privacy and confidentiality when working with clients from their community.

The broad demographic categories typically used in population-level data collection obstruct the characteristics, experiences, and needs of Yazidi and other minority cultural groups who must select the “white” racial category. This study adds to the weight of calls for a critical reconsideration of racial categorization frameworks widely used for demographic reporting in health research [68].

### Study limitations

This study has several limitations. First, personal bias may be introduced when one is familiar with the population, as was the case with one researcher who is part of the Yazidi community. To mitigate potential bias, steps were taken to triangulate the data by including multiple and diverse relevant perspectives from local content experts. We took steps to increase the trustworthiness of our findings by undergoing an iterative process of data gathering, researcher review and summarization, participant review, revision, and finalization. Another limitation is a lack of generalizability due to the limited sample size and the exclusive focus on one group of refugees. Although the sample size is small, participants in this study were selected due to their personal experience and extensive familiarity with the Yazidi community, specifically in the context of accessing healthcare.

## Conclusion

This study finds that informational, resource, and social barriers impede access to healthcare for Yazidi refugees. These barriers contribute to experiences of tension and precarity, and also to a drive toward amelioration. Although this study centers on the experience of Yazidi refugees, the recommendations address barriers common to many refugee and immigrant groups. Community agencies, healthcare organizations, policymakers, and other stakeholders must work together to develop strategies to reduce systemic barriers to equitable care. Community representation in priority-setting and decision-making is essential to ensure relevance, acceptability, and utilization of developed strategies.

## Data Availability

Data that support the findings of this study are available on reasonable request from the corresponding author [MK]. The data are not publicly available due to them containing information that could compromise research participant privacy/consent.
